# The association of APOC4 polymorphisms with premature coronary artery disease in a Chinese Han population

**DOI:** 10.1186/s12944-015-0065-7

**Published:** 2015-06-28

**Authors:** Shun Xu, Jie Cheng, Nan-hong Li, Yu-ning Chen, Meng-yun Cai, Sai-sai Tang, Haijiao Huang, Bing Zhang, Jin-ming Cen, Xi-li Yang, Can Chen, Xinguang Liu, Xing-dong Xiong

**Affiliations:** Institute of Aging Research, Guangdong Medical University, Xin Cheng Avenue 1#, Songshan Lake, Dongguan, 523808 People’s Republic of China; Guangdong Provincial Key Laboratory of Medical Molecular Diagnostics, Dongguan, People’s Republic of China; Institute of Biochemistry & Molecular Biology, Guangdong Medical University, Zhanjiang, People’s Republic of China; Department of Cardiovascular Disease, The First People’s Hospital of Foshan, Foshan, People’s Republic of China; Department of Cardiovascular Disease, The Affiliated Hospital of Guangdong Medical University, Zhanjiang, People’s Republic of China

**Keywords:** *APOC4*, Single nucleotide polymorphism, Premature coronary artery disease, Risk

## Abstract

**Background:**

Hypercholesterolemia arising from abnormal lipid metabolism is one of the critical risk factors for coronary artery disease (CAD), however the roles of genetic variants in lipid metabolism-related genes on premature CAD (≤60 years old) development still require further investigation. We herein genotyped four single nucleotide polymorphisms (SNPs) in lipid metabolism-related genes (rs1132899 and rs5167 in *APOC4*, rs1801693 and rs7765781 in *LPA*), aimed to shed light on the influence of these SNPs on individual susceptibility to early-onset CAD.

**Methods:**

Genotyping of the four SNPs (rs1132899, rs5167, rs1801693 and rs7765781) was performed in 224 premature CAD cases and 297 control subjects (≤60 years old) using polymerase chain reaction-ligation detection reaction (PCR–LDR) method. The association of these SNPs with premature CAD was performed with SPSS software.

**Results:**

Multivariate logistic regression analysis showed that C allele (OR = 1.50, *P* = 0.027) and CC genotype (OR = 2.84, *P* = 0.022) of *APOC4* rs1132899 were associated with increased premature CAD risk, while the other three SNPs had no significant effect. Further stratified analysis uncovered a more evident association with the risk of premature CAD among male subjects (C allele, OR = 1.65, and CC genotype, OR = 3.33).

**Conclusions:**

Our data provides the first evidence that *APOC4* rs1132899 polymorphism was associated with an increased risk of premature CAD in Chinese subjects, and the association was more significant among male subjects.

**Electronic supplementary material:**

The online version of this article (doi:10.1186/s12944-015-0065-7) contains supplementary material, which is available to authorized users.

## Background

Coronary artery disease (CAD) is the predominant cause of death and morbidity worldwide. Previous studies and clinical trials have established numerous environmental factors contributing to the development of CAD [1], such as obesity, hypercholesterolemia, alcohol intake, smoking, diabetes and hypertension [2]. Among the above risk factors, hypercholesterolemia arising from abnormal lipid metabolism has been considered to be one of the most key risk factors for CAD pathogenesis [3, 4]. Moreover, apart from these modifiable factors, accumulating evidences have demonstrated close associations of genetic variants or polymorphisms in candidate genes with the risk of CAD, especially the early-onset CAD [5–7].

Age is a critical contributing factor for the risk of CAD, age-related functional impairment including weak immune and inflammatory system, and other mechanisms involved in the regulation of the flow in the coronary arteries, exerted crucial effects during CAD development [8]. Thus, in order to exclude the effects of age-related dysfunction of CAD associated mechanisms, the association analysis of these four SNPs in lipids metabolism-related genes with the risk of CAD was performed only among the early-onset CAD subjects. Nonetheless, the definition for early-onset CAD patients discriminating from late-onset CAD is unspecific. In this study, we collected the CAD cases and control subjects younger than 60 years old, according to several previous published studies [9, 10].

Apolipoprotein C-IV (*APOC4*), a highly conserved lipid-binding protein belonging to the apolipoprotein C family, exerts a critical role in lipid metabolism [11, 12]. The *APOC4* gene is located on the chromosome 19q12-13.2, constructs the APOE/C1/C4/C2 gene cluster with apolipoprotein CI (*APOC1*), apolipoprotein CII (*APOC2*) and apolipoprotein E (*APOE*) [13], which is closely associated with plasma lipid levels, atherosclerotic plaque formation, and thus with the development of coronary artery disease and alzheimer’s disease [14, 15]. APOC4 is predominantly associated with very low-density lipoprotein (VLDL), and plays an important role in triglyceride metabolism [15]. Overexpression of apolipoprotein C-IV in transgenic mice can cause hypertriglyceridemia [16]. Moreover, previous study has revealed a significant association of rs5167 (Leu96Arg) and rs1132899 (Leu36Pro) polymorphisms in *APOC4* gene with triglyceride levels in women [17]. In addition, the T allele of APOC4 rs5167 polymorphism conferred modest effects on high density lipoprotein cholesterol (HDL-C) and apoAI levels [15]. Thus it was reasonable to speculate that *APOC4* polymorphisms might probably play an important role in CAD pathogenesis through impacting on plasma lipid profile.

Lipoprotein (*LPA*), also refers to as lipoprotein (a), a large glycoprotein attached to the low-density lipoprotein (LDL)-like particles, has been demonstrated to be associated with the risk of coronary artery disease [18]. LPA is highly homologous with plasminogen, can bind to the lysine sites available for plasminogen on the surface of fibrin, and thereby compete with plasminogen to interfere with the fibrinolytic process and inhibit its activation, which increases the formation of thrombosis, and finally promotes the atherogenesis and coronary artery disease pathogenesis [19, 20]. Additionally, the elevated LPA levels and corresponding genotypes were associated with increased risk of aortic valve stenosis (AVS) [21]. Genome-wide association study (GWAS) has revealed that the SLC22A3-LPAL2-LPA gene cluster was a risk locus for coronary artery disease, and the chromosomal region (6p26) where the SLC22A3-LPAL2-LPA gene cluster located is closely associated with the risk of CAD, and the LPA locus on 6q26-27 encoding lipoprotein has the strongest association [22]. Recent studies illustrated that the G allele of rs10455872, and the C allele of rs3798220 in *LPA* gene significantly elevated the LPA levels in peripheral blood, and the two SNPs in *LPA* were demonstrated to be the critical risk factors for CAD development, indicating that *LPA* polymorphisms were associated with CAD risk [23]. We herein focused on two other *LPA* polymorphisms (rs1801693 and rs7765781) in its exons, in order to investigate into the associations of these two SNPs with premature CAD risk.

Single nucleotide polymorphisms (SNPs) have been established to influence individual susceptibility for diverse human diseases. Accumulating evidences have suggested that SNPs within the lipid metabolism-related genes might potentially contribute to CAD [24–27]. Nonetheless, the genetic causes and underlying molecular mechanisms of these candidate genes for CAD require to be elucidated. Since aging effects, including weak immune system and relative high level exposure to environmental risk factors, rather than direct genetic effects, contribute to the risk of CAD in older subjects. Thus, we herein conducted a case–control study to investigate the association of the four SNPs in the lipid metabolism-related genes (rs5167 and rs1132899 in *APOC4*, rs1801693 and rs7765781 in *LPA*) with the risk of premature CAD (≤60 years old). Our data revealed that the C allele of rs1132899 in *APOC4* has a significant association with an increased risk of premature CAD in a Chinese population.

## Methods

### Study subjects

Two hundred twenty-four CAD patients and 294 control subjects (age ≤ 60 years old) were consecutively recruited from the First People’s Hospital of Foshan (Foshan, China) and the Affiliated Hospital of Guangdong Medical University (Zhanjiang, China) from March 2011 to February 2013. All the patients were newly diagnosed and previously untreated. Coronary angiography was initially performed to clarify the origin of chest pain or electrocardiographic abnormalities at rest or during exercise test. The diagnosis of CAD was confirmed by coronary angiography performed with the Judkins technique using a quantitative coronary angiographic system. CAD was defined as angiographic evidence of at least one segment of a major epicardial coronary artery with more than 50 % organic stenosis. Two cardiologists who were responsible for the assessment of angiograms both underwent strict training and complied with the same diagnostic criteria. Subjects with a history of hematologic, neoplastic, renal, liver, or thyroid diseases were excluded.

All subjects enrolled in this study were genetically unrelated ethnic Han Chinese. Each subject was interviewed to collect information on demographic data and risk factors related to CAD after obtaining the informed consent. The study was approved by the Medical Ethics Committee of the First People’s Hospital of Foshan and the Affiliated Hospital of Guangdong Medical University.

### Biochemical parameters analysis

The blood sample drawn from each subject was centrifuged at 2000 × g for 15 min immediately after collection and stored at −80 °C. The levels of plasma total cholesterol (TC), triglyceride (TG), high density lipoprotein cholesterol (HDL-C), and low density lipoprotein cholesterol (LDL-C) were measured enzymatically using a chemistry analyzer (Olympus, Japan). Glucose was analyzed by the glucose oxidase method with an Abbott V/P Analyzer (Abbott Laboratories, USA).

### DNA extraction

Genomic DNA was extracted from peripheral whole blood by TIANamp blood DNA extraction kit (TianGen Biotech, Beijing, China) according to the manufacturer’s instructions. All DNA samples were dissolved in water and stored at −20 °C until use.

### Genotyping

SNPs genotyping were performed utilizing polymerase chain reaction-ligase detection reaction (PCR-LDR) method (Shanghai Biowing Applied Biotechnology Company), as described in our previous study [28]. The sequence of primers and probes were listed in Additional file 1: Table S1.

### Statistical analysis

All the three SNPs were tested for confirmation with Hardy-Weinberg expectations by a goodness-of-fit χ^2^ test among the control subjects. Quantitative variables were expressed as mean ± standard deviation (SD), and qualitative variables were expressed as percentages. The differences of the demographic characteristics between the cases and controls were estimated using the χ^2^ test (for categorical variables) and Student’s *t* test (for continuous variables).

Multivariate association analyses with CAD risk, genotype frequencies were assessed by means of multivariate methods based on logistic regression analysis, the odds ratios (ORs) and 95 % confidence intervals (CIs) for the effect of SNPs on CAD risk adjusted by age, sex, smoking, drinking, hypertension, diabetes and hyperlipidemia. Association analyses between SNPs and blood lipid profiles were performed by one-way analysis of variance (ANOVA). The statistical analyses were performed using the SPSS software (version 21). A *P* value of less than 0.05 was used as the criterion of statistical significance.

## Results

### Characteristics of the study population

The characteristics of CAD cases and control subjects were listed in Table 1. In the lipid profiles comparison, TG and LDL-C were significantly higher in CAD patients than that in controls (*P* < 0.001, *P* < 0.001, respectively), whereas serum HDL-C levels were significantly higher among controls (*P* < 0.001), and no statistically significant difference between cases and controls was observed in TC levels (*P* = 0.160). Besides, the average fasting plasma glucose (FPG) in CAD cases was significantly higher than in controls (*P* < 0.001). CAD cases had higher levels of systolic blood pressure, diastolic blood pressure; the prevalence of smokers, alcohol consumers, and individuals with hypertension, diabetes or hyperlipidemia was significantly higher among the CAD patients. In addition, the number of female subjects in CAD cases was much lower than the male subjects. In all, these data demonstrated that male gender, smoking, alcohol intake, hypertension, hyperlipidemia and diabetes mellitus were the critical risk factors for premature CAD development in Chinese population.

**Table 1:** The characteristics of early-onset CAD cases and controls

Variable	Controls (*n* = 297)	Cases (*n* =224)	*P*-value^a^
Age (years)	50.49 ± 6.85	51.66 ± 6.47	**0.048** ^**b**^
Sex (male)	171 (57.6 %)	177 (79.0 %)	**<0.001**
Smoking	86 (29.0 %)	140 (62.5 %)	**<0.001**
Drinking	47 (15.8 %)	71 (31.7 %)	**<0.001**
Hypertension	82 (27.6 %)	142 (63.4 %)	**<0.001**
Diabetes	45 (15.2 %)	115 (51.3 %)	**<0.001**
Hyperlipidemia	123 (41.4 %)	169 (75.4 %)	**<0.001**
Systolic BP (mm Hg)	129.44 ± 16.17	141.68 ± 18.88	**<0.001**
Diastolic BP (mm Hg)	72.96 ± 9.91	76.87 ± 10.80	**<0.001**
FPG (mmol/L)	5.85 ± 2.36	6.67 ± 1.58	**<0.001**
Triglycerides (mmol/L)	1.60 ± 0.96	2.19 ± 1.09	**<0.001**
Total cholesterol (mmol/L)	4.64 ± 1.19	4.79 ± 1.27	0.160
LDL cholesterol (mmol/L)	1.45 ± 0.89	1.18 ± 0.34	**<0.001**
HDL cholesterol (mmol/L)	2.63 ± 0.99	3.08 ± 0.94	**<0.001**

^a^Two-sided chi-square test or independent-samples *t*-test

^b^ *P* values under 0.05 were indicated in bold font

### Multivariate associations of four SNPs with the risk of premature CAD

Four SNPs (rs5167 and rs1132899 in *APOC4*, rs1801693 and rs7765781 in *LPA*) were genotyped in 224 CAD patients and 294 control subjects (≤60 years old). The primary information for rs5167, rs1132899, rs1801693 and rs7765781 polymorphisms was listed in Table 2. Minor allele frequency (MAF) of all three SNPs in our controls was similar to MAF for Chinese in HapMap database (Table 2). All the genotype frequency distributions of the three SNPs in our control subjects followed Hardy-Weinberg equilibrium proportions (all *P* values ≥ 0.10, Table 2).

**Table 2:** Primary information for rs1132899, rs5167, rs6687605 and rs13306731 SNPs

Genotyped SNPs	rs1132899	rs5167	rs1801693	rs7765781
Chr Pos (Genome Build 104.0)	45448036	45448465	160969629	161007496
Gene	APOC4 Exon 2	APOC4 Exon 3	LPA Exon32	LPA Exon26
MAF^a^ for Chinese(CHB) in HapMap	0.244	0.366	0.439	0.489
MAF in our controls (*n* = 650)	0.328	0.481	0.449	0.359
*P* Value for HWE^b^ test in our controls	0.597	0.507	0.813	0.838

^a^MAF: minor allele frequency

^b^HWE: Hardy–Weinberg equilibrium

The allele and genotype distributions of the four SNPs in the cases and the controls were shown in Table 3. From the allelic association analysis, we found only rs1132899 showed statistical significance, and C allele was associated with a significantly increased risk of CAD (OR = 1.50, 95 % CI = 1.05–2.14, *P* = 0.027, Table 3). In addition, compared to TT genotype, the CC genotype exhibited an increased risk of CAD as well (OR = 2.84, 95 % CI = 1.17–6.92, *P* = 0.022, Table 3). These data indicated that *APOC4* SNP rs1132899 was associated with premature CAD risk (age ≤ 60 years old), and that individuals carrying C allele might have significantly increased CAD susceptibility. However, we did not find any association between rs5167, rs1801693 and rs7765781 and the risk of CAD (Table 3).

**Table 3:** Multivariate associations of the SNPs in *APOC4* and *LPA* gene with the risk of premature CAD

Type	Controls (*n* = 297)	Cases (*n* = 224)	OR (95 % CI)^a^	*P*-value^a^
	No. (%)	No. (%)		
APOC4 rs1132899				
T	195 (32.8)	132 (29.5)	1.00	-
C	399 (67.2)	316 (70.5)	1.50 (1.05–2.14)	**0.027** ^**b**^
TT	30 (10.1)	15 (6.7)	1.00	-
CT	135 (45.5)	102 (45.5)	2.15 (0.89–5.20)	0.089
CC	132 (44.4)	107 (47.8)	2.84 (1.17–6.92)	**0.022**
APOC4 rs5167				
T	286 (48.1)	217 (48.4)	1.00	-
G	308 (51.9)	231 (51.6)	0.75 (0.54–1.03)	0.078
TT	66 (22.2)	47 (21.0)	1.00	-
GT	154 (51.9)	123 (54.9)	1.35 (0.78–2.35)	0.289
GG	77 (25.9)	54 (24.1)	1.79 (0.94–3.43)	0.078
LPA rs1801693				
T	327 (55.1)	249 (55.6)	1.00	-
C	267 (44.9)	199 (44.4)	0.94 (0.70–1.28)	0.707
TT	89 (30.0)	71 (31.7)	1.00	-
CT	149 (50.2)	107 (47.8)	1.00 (0.61–1.65)	0.997
CC	59 (19.8)	46 (20.5)	1.14 (0.62–2.10)	0.681
LPA rs7765781				
C	213 (35.9)	159 (35.5)	1.00	-
G	381 (64.1)	289 (64.5)	1.04 (0.76–1.43)	0.807
CC	39 (13.1)	28 (12.5)	1.00	-
CG	135 (45.5)	103 (46.0)	1.16 (0.59–2.25)	0.674
GG	123 (41.4)	93 (41.5)	1.13 (0.58–2.22)	0.724

^a^Adjusted for age, sex, smoking, drinking, hypertension, diabetes and hyperlipidemia

^b^ *P* values under 0.05 were indicated in bold font

### Stratification analyses of *APOC4* rs1132899 polymorphism and risk of CAD

We further evaluated the alleles or genotypes of *APOC4* rs1132899 and CAD susceptibility after stratifying the subjects by sex, status of smoking or drinking. Stratification analyses by sex revealed that the increased risk of CAD was more evident among male subjects carrying C allele (OR = 1.65, 95 % CI = 1.06–2.58, *P* = 0.028, Table 4) or the CC genotype (OR = 3.33, 95 % CI =1.14–9.69, *P* = 0.027, Table 4). In addition, only the CC genotype of rs1132899 polymorphism exhibited an association with enhanced risk of CAD in smokers (OR = 3.59, 95 % CI =1.08–12.0, *P* = 0.037, Table 4), but not in non-smokers. No more evident association between *APOC4* rs1132899 polymorphism and risk of CAD was observed among subgroups by status of drinking (data not shown).

**Table 4:** Multivariate associations of the rs1132899 in *APOC4* gene with the risk of CAD by further stratification for sex and smoking status

	Male		Smokers	
Genotype	OR (95 % CI)^a^	*p*-value^a^	OR (95 % CI)^b^	*P*-value^b^
T	1.00	**-**	1.00	**-**
C	1.65 (1.06–2.58)	**0.028** ^**a**^	1.58 (0.93–2.70)	0.094
TT	1.00	**-**	1.00	**-**
CT	2.29 (0.80–6.57)	0.123	3.07 (0.95–9.96)	0.061
CC	3.33 (1.14–9.69)	**0.027** ^**c**^	3.59 (1.08–12.0)	**0.037**

^a^Adjusted for age, smoking, drinking, hypertension, diabetes and hyperlipidemia

^b^Adjusted for age, sex, drinking, hypertension, diabetes and hyperlipidemia

^c^ *P* values under 0.05 were indicated in bold font

### Multivariate associations of the *APOC4* SNPs with the lipid profile

In order to probe into the potential explanation to the enhanced effects of *APOC4* rs1132899 polymorphism on early-onset CAD risk (≤60 years old), we further analyzed the association between *APOC4* rs1132899 polymorphism and LDL-C, HDL-C, TC and TG levels. However none of the above lipids profile exhibited significant association with *APOC4* rs1132899 polymorphism (data not shown). Nonetheless, CC genotype of rs1132899 conferred about 0.09 mmol/L increase in triglyceride (TG) levels compared to CT/TT genotype (1.95 mmol/L vs 1.86 mmol/L) in male subjects compared to CT/TT genotype, though the difference was non-significant (Table 5). Thus, these results indicated that, in addition to influence the lipid profile, there might be other mechanisms contributing the increased risk of *APOC4* rs1132899 polymorphism in premature CAD, which still require further analysis in a larger sample size.

**Table 5:** ANOVA analysis of the association between rs1132899 in *APOC4* gene and the LDL-C, HDL-C, TC and TG levels by further stratification for sex

Variable	Male				Female	
	CC	CT + TT	*P*-value^a^	CC	CT + TT	*P*-value^a^
Triglycerides (mmol/L)	1.95 ± 1.13	1.86 ± 1.08	0.457^a^	1.68 ± 084	1.81 ± 1.03	0.367
Total cholesterol (mmol/L)	4.65 ± 1.20	4.67 ± 1.23	0.870	4.82 ± 1.07	4.74 ± 1.41	0.668
LDL cholesterol (mmol/L)	2.83 ± 1.02	2.81 ± 0.87	0.882	2.83 ± 1.02	2.83 ± 1.16	0.996
HDL cholesterol (mmol/L)	1.34 ± 1.17	1.22 ± 0.34	0.191	1.42 ± 0.35	1.45 ± 0.48	0.609

^a^Two-sided chi-square test or independent-samples *t*-test

## Discussion

The fundamental pathogenesis of CAD is the dysfunction of lipid metabolism, and thus the disorder of plasma lipid profile, which caused by both individual’s genetic makeup and various environmental factors. Previous studies have demonstrated the effects of *APOC4* and *LPA* in abnormal blood lipids including hypercholesterolemia or hypertriglyceridemia, indicating the potential role of *APOC4* and *LPA* in the CAD development. Nonetheless, the association between SNPs in *APOC4* and *LPA* gene and CAD risk still required to be fully elucidated. The potential risk of CAD in older subjects is more likely due to the aging effects as weak immune system and relative high level exposure to environmental risk factors, rather than direct genetic effects. Thus, in this study, we performed a genetic association analysis on the four SNPs (rs1132899 and rs5167 in *APOC4*, rs1801693 and rs7765781 in *LPA*) among early-onset CAD subjects (≤60 years old). Our data revealed that the *APOC4* rs1132899 polymorphism was associated with increased risk of both premature CAD, and the association was more remarkable among male subjects. Taken together, our study suggested that *APOC4* polymorphisms might play an important role in the early-onset CAD pathogenesis.

The APOE/C1/C4/C2 gene cluster has been established to be a critical region for several human diseases, including CAD, and thus the polymorphisms of the locus have been considered to be closely associated with the risk of CAD. However, the effects of *APOC4* rs1132899 and rs5167 polymorphisms on CAD risk are still unknown. Kamboh *et al.* has reported that the rs1132899 and rs5167 polymorphisms were significantly associated with triglyceride levels in women; moreover, *APOC4* rs5167 polymorphism exerted a modest effect on HDL-C levels. Hypertriglyceridemia and hypercholesterolemia are crucial risk factors for atherosclerosis, CAD and other arterial cardiovascular diseases, which indicated the important role of the two *APOC4* polymorphisms during CAD development. Our data found that rs1132899 endowed C allele carriers with significant increased CAD risk, which is in consistent with the results from the above association analysis between the two polymorphisms and plasma lipids levels.

Our stratified analyses revealed that the increased risk of *APOC4* rs1132899 polymorphism in CAD was more evident among male subjects, whereas no significant association was observed from the female group (Table 4). The potential explanation to the gender variance is as follow: 1. Previous studies have unraveled that the most common risk factor in young men was cigarette smoking, while the major risk factor was hypercholesterolaemia in young women [29]. Our data showed that the rs1132899 polymorphism only exhibited an association with enhanced risk of CAD in smokers, but not in non-smokers, which was consistent with the association in male subjects (Table 5); 2. The stratified analyses showed that the CC genotype of rs1132899 modestly increased the triglyceride (TG) levels compared in male subjects, whereas female subjects carrying CC genotype exhibited mildly decreased TG levels; 3. Sex hormones play an important role in coronary artery disease, a proper ratio of estrogen:testosterone is essential during CAD development s [30], which might account for the sex variance of rs1132899 polymorphism for the risk of CAD.

Several limitations in this case–control study still need to be addressed. First, the case subjects and controls enrolled from hospitals may not represent the general population. However, the genotype distribution of the controls was in Hardy-Weinberg equilibrium. Second, the moderate sample size limited the statistical power of our study, especially for the case subjects. Finally, further studies in different population could help to further verify the significance of the association between the rs1132899 polymorphism and the risk of early-onset CAD. However, our results provided valuable insights and interesting information and might serve to guide future studies in this area.

## Conclusions

In aggregate, our study firstly uncovered that the C allele of *APOC4* rs1132899 was associated with an increased risk of early-onset CAD in a Chinese population, and the association is more evident among male subjects, which potentially due to the modestly elevated TG levels. Further studies with larger sample size and in diverse ethnic populations are required to confirm the general validity of our findings.

## Additional file

Additional file 1: Table S1. The sequences of the primers and probes used to genotype SNPs.

## Abbreviations

APOC4: Apolipoprotein C-IV
LPA: Lipoprotein
CAD: Coronary artery disease
SNP: Single nucleotide polymorphism
PCR-LDR: Polymerase chain reaction-ligase detection reaction
OR: Odds ratio
CI: Confidence interval
LDL-C: Low density lipoprotein cholesterol
TC: Total cholesterol
TG: Triglyceride
HDL-C: High density lipoprotein cholesterol
