# Supplementary material for: The association of APOC4 polymorphisms with premature coronary artery disease in a Chinese Han population
Source: Lipids Health Dis. 2015 Jun 28;14:63. doi: 10.1186/s12944-015-0065-7 (PMC4511022; doi:10.1186/s12944-015-0065-7)
Supplement: Additional file 1: Table S1. — The sequences of the primers and probes used to genotype SNPs. [file 12944_2015_65_MOESM1_ESM.doc]

**Table S1 The sequences of the primers and probes used to genotype SNPs.**

| **Name** | **Sequence (5’-3’)** |
| --- | --- |
| **Primers** |  |
| rs1132899-F | CCTCTGGTTCCACCTAGCAT |
| rs1132899-R | CGACTCATCTTTAGCTTTGG |
| rs5167-F | CATGCAGACCTACTATGACG |
| rs5167-R | GTCTTTGGATTCGAGGAACC |
| rs1801693-F | TGCAGTACTCCCACCTGATG |
| rs1801693-R | AGGAATCCAGATGCCGATAC |
| rs7765781-F | TCACAACTCCCACGGTGGTC |
| rs7765781-R | TCTGACTGCAGGCTTCTTAC |
| **Probes** |  |
| rs1132899-FAM | P-GAGCCCCCCACCAAAGCTAAAGATGTTTTTTTTTTTTTTTTTTTTTTTTTTTTTTTTTTTTTTTTTTTTTTTTTTTTTT-FAM |
| rs1132899-C | TTTTTTTTTTTTTTTTTTTTTTTTTTTTTTTTTTTTTTTTTTTTTTTTTTTTTTTTTTTTTTTTTTTTAGCCAGAGGCCCAGGAAGGAACCCC |
| rs1132899-T | TTTTTTTTTTTTTTTTTTTTTTTTTTTTTTTTTTTTTTTTTTTTTTTTTTTTTTTTTTTTTTTTTTAGCCAGAGGCCCAGGAAGGAACCCT |
| rs5167-FAM | P-GCGGACCCAGGTCCCTCAGGTGGTCTTTTTTTTTTTTTTTTTTTTTTTTTTTTTTTTTTTTTTTTTTTTTTTTTTTTTT-FAM |
| rs5167-G | TTTTTTTTTTTTTTTTTTTTTTTTTTTTTTTTTTTTTTTTTTTTTTTTTTTTTTTTTTTTTTTTTTTTTTTTTTGATTCGAGGAACCAGGCCTTGGTGC |
| rs5167-T | TTTTTTTTTTTTTTTTTTTTTTTTTTTTTTTTTTTTTTTTTTTTTTTTTTTTTTTTTTTTTTTTTTTTTTTTTTTTGATTCGAGGAACCAGGCCTTGGTGA |
| rs1801693-FAM | P-TGGTAAAACACCAAGGGCCTGTATCTTTTTTTTTTTTTTTTTTTTTTTTTTTTTTTTTTTTTTTTTTTTTTTTTTTTTT-FAM |
| rs1801693-T | TTTTTTTTTTTTTTTTTTTTTTTTTTTTTTTTTTTTTTTTTTTTTTTTTTTTTTTTTTTTTTTTTACTCCCACCTGATGCTGGGGTCCA |
| rs1801693-C | TTTTTTTTTTTTTTTTTTTTTTTTTTTTTTTTTTTTTTTTTTTTTTTTTTTTTTTTTTTTTTTACTCCCACCTGATGCTGGGGTCCG |
| rs7765781-FAM | P-TTCCTTCTGAAGAAGGTAAGAAGCCTTTTTTTTTTTTTTTTTTTTTTTTTTTTTTTTTTTTTTTTTTTTTTTTTTTTTT-FAM |
| rs7765781-C | TTTTTTTTTTTTTTTTTTTTTTTTTTTTTTTTTTTTTTTTTTTTTTTTTTTTTTTTTTTTTTTTTTTTTTGTGGTCCCAGTTCCAAGCACAGAGG |
| rs7765781-G | TTTTTTTTTTTTTTTTTTTTTTTTTTTTTTTTTTTTTTTTTTTTTTTTTTTTTTTTTTTTTTTTTTTTTTTTGTGGTCCCAGTTCCAAGCACAGAGC |
